# Integrated genomic approaches identify upregulation of *SCRN1* as a novel mechanism associated with acquired resistance to erlotinib in PC9 cells harboring oncogenic EGFR mutation

**DOI:** 10.18632/oncotarget.7318

**Published:** 2016-02-11

**Authors:** Nayoung Kim, Ahye Cho, Hideo Watanabe, Yoon-La Choi, Meraj Aziz, Michelle Kassner, Je-Gun Joung, Angela KJ Park, Joshua M. Francis, Joon Seol Bae, Soo-min Ahn, Kyoung-Mee Kim, Joon Oh Park, Woong-Yang Park, Myung-Ju Ahn, Keunchil Park, Jaehyung Koo, Hongwei Holly Yin, Jeonghee Cho

**Affiliations:** ^1^ Department of NanoBio Medical Science, Dankook University, Cheonan 31116, Republic of Korea; ^2^ Department of Health Sciences and Technology, SAIHST, Sungkyunkwan University, Seoul 135-967, Republic of Korea; ^3^ Department of Medicine, Division of Pulmonary, Critical Care and Sleep Medicine, New York, NY 10029, USA; ^4^ Tisch Cancer Institute, Icahn School of Medicine at Mount Sinai, New York, NY 10029, USA; ^5^ Department of Pathology, Samsung Medical Center, Sungkyunkwan University School of Medicine, Seoul 135-967, Republic of Korea; ^6^ Cancer and Cell Biology Division, Translational Genomics Research Institute, Scottsdale, AZ 85259, USA; ^7^ Samsung Genome Institute, Samsung Medical Center, Seoul 135-967, Republic of Korea; ^8^ Cancer Program, Broad Institute of Harvard and MIT, Cambridge, MA 02142, USA; ^9^ Division of Hematology-Oncology, Department of Medicine, Samsung Medical Center, Sungkyunkwan University School of Medicine, Seoul 135-967, Republic of Korea; ^10^ Department of Brain and Cognitive Sciences, DGIST, Daegu 42988, Republic of Korea

**Keywords:** EGFR, SCRN1, lung adenocarcinoma, erlotinib resistance

## Abstract

Therapies targeting the tyrosine kinase activity of Epidermal Growth Factor Receptor (EGFR) have been proven to be effective in treating a subset of non-small cell lung cancer (NSCLC) patients harboring activating *EGFR* mutations. Inevitably these patients develop resistance to the EGFR-targeted tyrosine kinase inhibitors (TKIs). Here, we performed integrated genomic analyses using an *in vitro* system to uncover alternative genomic mechanisms responsible for acquired resistance to EGFR-TKIs. Specifically, we identified 80 genes whose expression is significantly increased in the erlotinib-resistant clones. RNAi-based systematic synthetic lethal screening of these candidate genes revealed that suppression of one upregulated transcript, SCRN1, a secernin family member, restores sensitivity to erlotinib by enhancing inhibition of PI3K/AKT signaling pathway. Furthermore, immunohistochemical analysis revealed increased levels of SCRN1 in 5 of 11 lung tumor specimens from EGFR-TKIs resistant patients. Taken together, we propose that upregulation of *SCRN1* is an additional mechanism associated with acquired resistance to EGFR-TKIs and that its suppression serves as a novel therapeutic strategy to overcome drug resistance in these patients.

## INTRODUCTION

The success of genome-directed small molecule inhibitors targeted against aberrantly activated tyrosine kinases have changed the clinical paradigm of cancer treatments and ushered in the age of precision medicine [1, 2]. In particular, epidermal growth factor receptor (EGFR)-targeted therapy with tyrosine kinase inhibitors (TKIs) such as erlotinib, gefitinib and afatinib have been effective in a subset of patients with non-small cell lung cancer (NSCLC) harboring *EGFR* activating mutations [3–6]. Two common *EGFR* somatic alterations, the L858R mutation in exon 21 and exon 19 in-frame deletions encompassing amino acids 747 to 749, represent about 90% of *EGFR* mutations in lung adenocarcinoma, and predict clinical responses to EGFR-TKIs [7–12]. Dramatic radiologic responses are observed with the EGFR-TKIs, however, almost all patients become resistant less than 1 year after initial treatment [13]. The most prevalent mechanism of acquired resistance, accounting for ∼50% of resistant cases, is the acquisition of a secondary *EGFR* mutation, a substitution of threonine at the “gatekeeper” amino acid 790 to methionine (T790M) in exon 20, resulting in increased binding affinity of EGFR to ATP over inhibitors [14–16].

In addition to the *EGFR* gatekeeper mutation, altered expression profiles, somatic single nucleotide variants and copy number alterations have also been found as mechanisms driving acquired resistance [17, 18]. These include gene amplification of *MET*, *ERBB2* or *CRKL* [19–21], somatic mutations in *PI3KCA* or *BRAF* [22, 23], *NF1* loss [24], and increased levels of IGF1R or AXL [25, 26]. Furthermore, epithelial-to-mesenchymal transition (EMT) or histological transformation to small-cell lung cancer has been reported to be responsible for EGFR-TKIs resistance [27]. Nevertheless, the mechanism of acquired resistance is still unknown for about 30% of remaining cases [28, 29].

In the present study, we carried out integrated genomic analyses to identify additional genomic alterations associated with acquired EGFR-TKIs resistance, and in particular, to discover resistance mechanisms that occur in the context of enhanced enzymatic activity associated with mutant EGFR. Therefore we established an erlotinib-resistant *in vitro* model system using PC9 NSCLC cells ectopically overexpressing the exon 19 deletion *EGFR* mutant and identified genes whose expression is significantly increased or decreased in erlotinib-resistant clones compared to parental cell lines by expression profiling. Utilizing further RNAi-based synthetic lethal screening, we found that suppression of *SCRN1* in erlotinib-resistant clones restores drug sensitivity, suggesting that upregulation of *SCRN1* may be a new mechanism for rendering the *EGFR* mutant-lung cancer cell lines to erlotinib resistance.

## RESULTS AND DISCUSSION

### Establishment and characterization of a model for overexpressed EGFR-mediated mechanism of EGFR-TKIs resistance in lung adenocarcinoma cell line

Oncogenic *EGFR* mutations in NSCLC patients are of significant clinical importance, however, the role that the elevated kinase activity associated with mutant EGFR is largely unexplored. To address this uncertainty, we sought to examine: 1) if increased kinase acitivity promotes the onset of acquired resistance to EGFR tyrosine kinase inhibitor erlotinib and 2) how it contributes to resistance mechanisms. We first generated a stable *EGFR* mutant overexpression cell model system using PC9 lung adenocarcinoma cells which harbor an endogenous *EGFR* exon 19 deletion (Ex19Del) mutation and are sensitive to either erlotinib or gefitinib [30]. To specifically investigate the role of elevated enzymatic activity of Ex19Del mutant in EGFR-TKI resistance, and not be confounded by constitutive phosphorylation-mediated downstream signaling, we utilized a phosphorylation-impaired EGFR mutant. In this particular experimental setup, all 10 C-terminal tyrosine residues were substituted to phenylalanine in the background of exon 19 deletion mutant (Ex19Del/CYF10) in generating the cell model.

**Figure 1:**
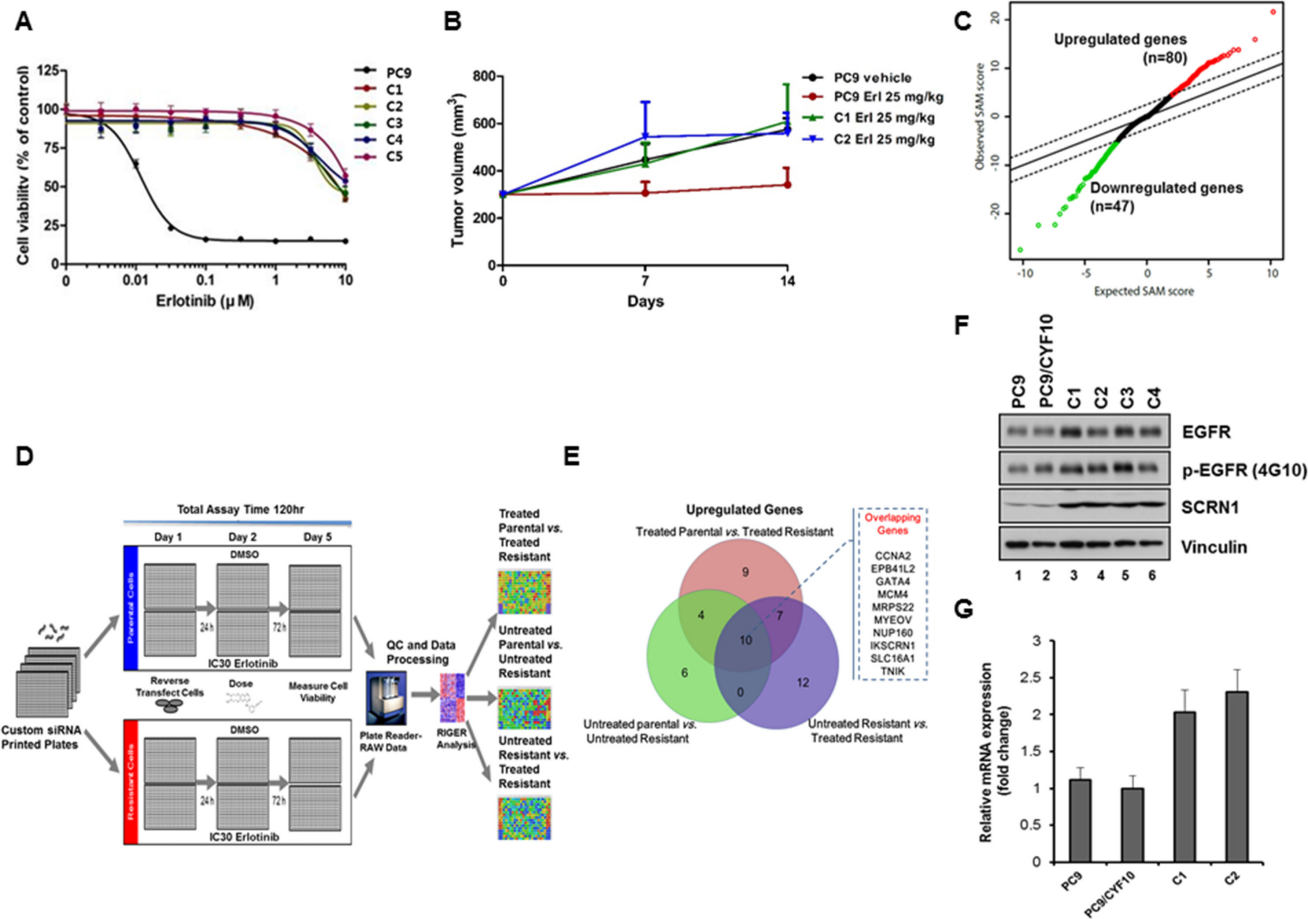
Identification of SCRN1 upregulation as a potential erlotinib resistant gene by RNAseq analysis followed by siRNA synthetic lethality screening (**A**) Growth of five isolated resistant PC9/CYF10 cell clones (C1–C5) is unaffected with erlotinib treatment. The results are presented as a mean ± SD of sextuplicate wells and are representative of three independent experiments. (**B**) Xenograft of erlotinib resistant clones generate tumor and remain refractory to erlotinib treatment. (**C**) Rank-ordered by statistical SAM score for differential expression in erlotinib-resistant PC9/CYF10 cells compared to erlotinib-sensitive parental cells from RNA-seq data and plotted against expected SAM score. A total of 21,514 genes are plotted. Red circles indicate significantly upregulated genes (*n* = 80) in erlotinib resistant cells with a score that deviates from expected distribution at delta slope of 2.5. Green circles indicate significantly downregulated genes (*n* = 47) in erlotinib resistant cell lines. (**D**) Schematic of siRNA synthetic lethality loss-of-function screen measuring cell viability in the presence or absence of the erlotinib. (**E**) Overlapping hits selected from data analysis of siRNA screening in three conditions are shown in the Venn diagram. The 10 overlapping genes among the three conditions are listed in the Figure (**F**) The levels of SCRN1 protein are significantly elevated in all erlotinib-resistant clones compared to parental control cells as shown by immunoblot analysis. Vinculin serves as a loading control. (**G**) Quantitative RT-PCR for *SCRN1* in parental and resistant clones validated that mRNA levels of *SCRN1* clones are higher in C1 and C2 than parental control cells. The fold change in *SCRN1* expression is shown in log2 in graph.

We then established erlotinib-resistance in the PC9 cell model by culturing in the presence of escalating doses of erlotinib from 0.05 μM to 10 μM, and then isolating individual single-cell clones, as previously described [19]. Notably, Ex19Del/CYF10 expressing PC9 (PC9/CYF10) cells acquired the resistance to erlotinib much faster than PC9 parental (51 days vs. 151 days), demonstrating that increased enzymatic activity of mutant EGFR by overexpression of mutant EGFR lacking autophosphorylation promotes the acquisition of erlotinib resistance in PC9 cells. The resistance of single-cell derived PC9/CYF10 clones (C1–C5) to erlotinib was further confirmed by cell viability (Figure 1A), colony formation assays in soft agar (Supplementary Figure S1A) as well as *in vivo* subcutaneous mouse xenografts (Figure 1B). Immunoblotting analysis revealed that when compared to the PC9/CYF10 parental cell line, the phosphorylation of endogenous EGFR as well as its downstream signaling molecules, AKT and ERK1/2 in clone 1 (C1) and clone 2 (C2) cells, were not completely inhibited by erlotinib treatment (Supplementary Figure S1B). In contrast to previous studies that reported the emergence of *EGFR* T790M gatekeeper secondary mutation in PC9 cells [31, 32], we detected no additional mutation on *EGFR* in this context (Supplementary Figure S1C) [25, 33–35]. In addition, genomic alterations previously identified to be associated with drug resistance including increased levels of ErbB2, AXL, MET, NF-κB, Vimentin or decreased E-cadherin or PTEN loss [13] were not detected by immunoblotting analysis in the erlotinib-resistant cell clones (Supplementary Figure S1D). Therefore, in this model, the incomplete inhibition of EGFR signaling by erlotinib and acquired erlotinib resistance is likely induced through a previously uncharacterized mechanism(s) [21, 36] and thus ideally suited to explore novel genomic alterations mediating resistance to erlotinib in the context of increased EGFR oncogenic activity without receptor autophosphorylation.

### RNAseq analyses identified gene signature of erlotinib-resistance in lung adenocarcinoma cell line

RNAseq-based expression profiles of two resistant clones (C1 and C2) and two parental cell lines with or without erlotinib treatment were generated to identify differentially expressed genes that may be associated with erlotinib resistance (Figure 1C and Supplementary Table S1 and Supplementary Figure S2A). To nominate candidate genes whose increased expression levels may be responsible for acquired resistance to erlotinib for further functional screening, we filtered out the genes with low expression as well as whose effect size of difference was minimal (see Materials and Methods for detail). In addition, we excluded the genes whose expression simply increased in response to erlotinib treatment, by comparing expression of parental PC9 cells after treatment with erlotinib. Upon these analyses, 80 genes were selected whose expression is significantly upregulated in erlotinib-resistant PC9/CYF10 cells (C1 and C2) and 47 genes that were downregulated (Figure 1C and Supplementary Table S2 and Supplementary Figure S2A). Notably, gene set enrichment analysis (GSEA) revealed that the down-regulated genes are significantly correlated with the down-regulated expression signature found in erlotinib-resistant NCI-H1975 cells upon treatment with an irreversible EGFR-TKI, which have both L858R and T790M mutations in *EGFR* [37]. These results are consistent with our hypothesis that these cell lines may have expression changes reversible by reactivation of certain transcriptional programs (Supplementary Figure S2B).

### siRNA synthetic lethality screening identified genes associated with resistance to EGFR-TKIs

Next, to investigate whether any of the 80 upregulated candidate genes may function as novel genomic determinants of acquired erlotinib resistance, we performed synthetic lethality screening with siRNAs targeting these genes in the C1 and C2 cells (Figure 1D). All expected performance parameters passed the screen quality control (QC) evaluation, revealing a low Coefficient of variation (CV) < 10%, siRNA high transfection efficiency of > 98% and high Z-factor of > 0.77. In addition, we observed high coefficient of determination of R^2^ values between the replicates within each run as well as the two biological runs for each cell lines (Supplementary Figure S2C and S2D). *GFP* was chosen as the siRNA control. We used the RIGER method [38] to determine the differential lethality in three different comparisons; erlotinib-treated resistant cells vs. erlotinib-treated parental cells, untreated resistant cells vs. untreated parental cells, and erlotinib-treated resistant cells vs. untreated resistant cells (Supplementary Table S3). Of the 48 statistically significant genes selected, 10 were commonly identified as potential modulators of survival of C1 and C2 cells in the three different comparisons (Figure 1E). Among those 10 genes, we found *SCRN1* to be of great interest because its upregulation was strongly associated with genomic alterations of *EGFR* across all three available lung adenocarcinoma data sets in cBioPortal (www.cbioportal.org) (Supplementary Table S4) [39, 40]. We confirmed that both SCRN1 protein and mRNA levels were significantly higher in resistant clones than in parental PC9/CYF10 cells (Figure 1F and 1G). Furthermore, SCRN1, a 50kDa cytosolic protein involved in the regulation of exocytosis from mast cells [41], is known to be upregulated in various cancers including gastric, prostate and colorectal cancers and its expression is correlated with poor prognosis of synovial sarcoma [42–45]. Thus, we decided to explore whether upregulation of *SCRN1* serve as a potential mechanism for EGFR-TKIs resistance by further functional characterization.

### SCRN1 overexpression is associated with EGFR-TKIs resistance and attenuates the effect of erlotinib on the downstream AKT pathway

**Figure 2:**
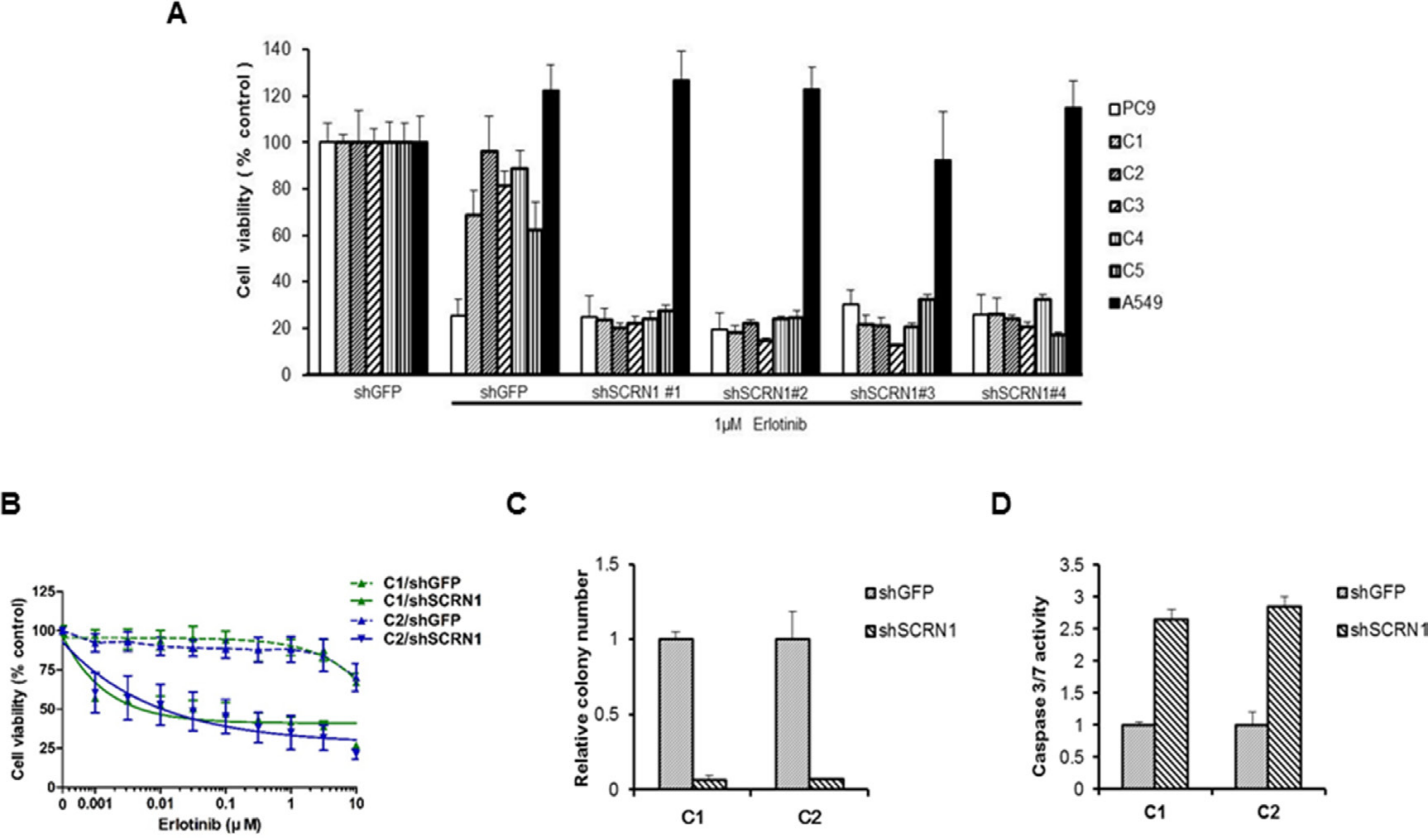
Downregulation of *SCRN1* in erlotinib-resistant cell clones enhanced the drug sensitivity and cellular apoptosis in response to erlotinib (**A**) Suppression of *SCRN1* by shRNA in erlotinib resistant clones increased erlotinib sensitivity. A549 cells were used as a negative control for the experiment. (**B**) Resistant clone C1 and C2 respond to erlotinib following *SCRN1* knockdown by shRNA in concentration dependent manner. The results are indicated as mean +/− SD of sextuplicate wells and are representative of three independent experiments. (**C**) C1 and C2 resistant clones are dependent on *SCRN1* for their transforming potential. The bar graph depicts the relative number of colonies in C1 or C2 transfected with sh*SCRN1* normalized to the number of colonies formed by cells transfected with shGFP (*n* = 3, mean + SD). (**D**) Knockdown of *SCRN1* increases caspase 3/7 activities in C1 and C2 clones. Values are the means + SD from three independent experiments.

We next investigated whether the increased *SCRN1* expression found in resistant clones plays a role in rendering erlotinib resistance. Specifically, we examined whether suppression of *SCRN1* via shRNAs (sh*SCRN1*) in the resistant clones leads to restoration of erlotinib sensitivity. All five erlotinib resistant clones (C1 to C5) transfected with four individual sh*SCRN1*, showed a reduction of cell viability upon erlotinib treatment at a comparable level to parental PC9 cells (Figure 2A), confirming downregulation of *SCRN1* resensitizes these clones to treatment. The same clones transduced with control shRNA (sh*GFP*) remained refractory to erlotinib (Figure 2A). In contrast, the growth of another lung adenocarcinoma cell line, A549 which harbors wild-type *EGFR*, was unaffected, suggesting that increased sensitization to erlotinib by shRNA-mediated suppression of *SCRN1* is specific to EGFR Ex19Del resistant cells (Figure 2A). In addition, growth inhibition of C1 and C2 cells in the cells with sh*SCRN1*, but not in the cells with sh*GFP* was dose-dependent (Figure 2B). Furthermore, we found that sh*SCRN1* expressing resistant clones exhibited decreased colony formation even in the absence erlotinib treatment when compared to cells stably expressing control sh*GFP* (Figure 2C), suggesting that SCRN1 may play a crucial role in oncogenic growth of these cells. This result is consistent with recent findings that suppression of SCRN1 in colon cancer cells inhibited cell proliferation and colony formation [44, 46]. Caspase 3/7 activity was markedly increased in resistant clones stably expressing sh*SCRN1*, indicating that this impaired transforming ability through down-regulation of *SCRN1* may be mediated via apoptosis (Figure 2D) [47]. Taken together, these data demonstrate that upregulation of *SCRN1* not only confers resistance but also is important for oncogenic activity in our erlotinib resistant clones, suggesting that suppression of SCRN1 may serve as an additional therapeutic strategy to overcome acquired erlotinib resistance.

**Figure 3:**
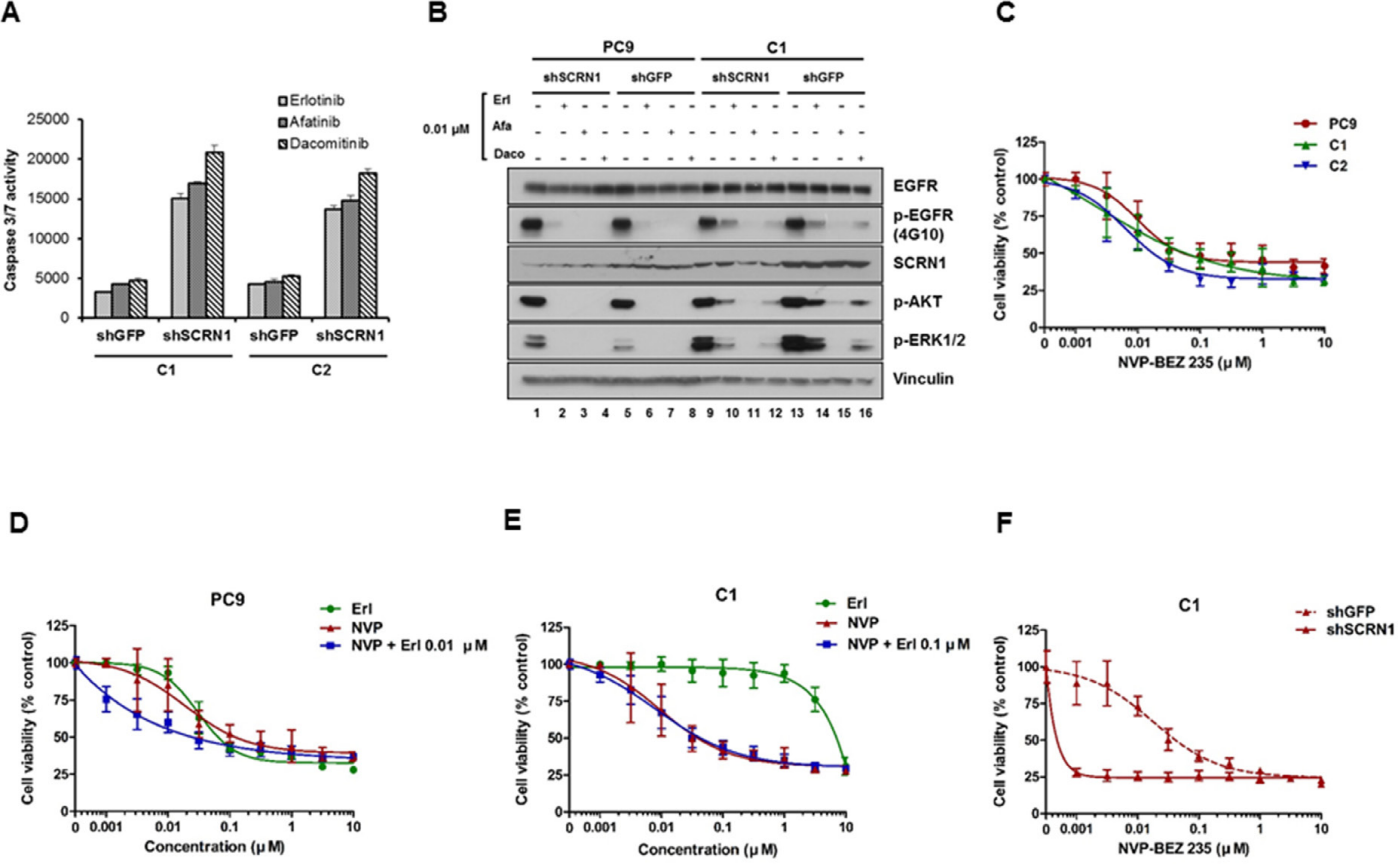
Activation of PI3K/AKT signaling pathways is essential for growth of erlotinib resistant cells (**A**) Caspase 3/7 activities in C1 and C2 cells treated with EGFR-TKIs were significantly enhanced following *SCRN1* knockdown compared to sh*GFP* control. Values are the means + SD from three independent experiments. (**B**) Levels of constitutively phosphorylated AKT and ERK1/2 were more robustly reduced by either erlotinib or dacomitinib in C1 cells transfected with shSCRN1 than in those with sh*GFP*. (**C**) Growth of C1 and C2 cells in presence of PI3K/AKT inhibitor NVP-BEZ235 is equivalent to that of PC9 parental cells. The results are presented as a mean ± SD of sextuplicate wells and are representative of three independent experiments. (**D** and **E**) Erlotinib synergistically increased the sensitivity of NVP-BEZ235 for PC9 cell (C), but not for C1 cells (D). The results are presented as a mean ± SD of sextuplicate wells and are representative of three independent experiments. (**F**) Growth of C1 cells was synergistically inhibited by NVP-BEZ235 in combination with shRNA-mediated silencing of SCRN1. The results are presented as a mean ± SD of sextuplicate wells and are representative of three independent experiments.

While suppression of *SCRN1* expression alone increased apoptosis, we observed that suppression of SCRN1 in combination with erlotinib treatment enhanced apoptotic activity (Figure 3A). In addition, this effect is larger following the treatment by the second generation irreversible EGFR-TKIs, afatinib or dacomitinib [6, 48], suggesting that downregulation of SCRN1 increases the drug efficacy of not only erlotinib but also the other EGFR-TKIs (Figure 3A).

Biochemical analyses show that levels of constitutively phosphorylated AKT and ERK1/2 were reduced by either erlotinib or dacomitinib in C1 cells transduced with sh*SCRN1* as compared to those with sh*GFP* (Figure 3B lanes 10, 12, 14 and 16, and Supplementary Figure S3A and S3B). In contrast, parental cells or resistant clones with sh*SCRN1* alone had little or no effect in attenuating AKT or ERK1/2 activation under normal cell culture conditions (Figure 3B; lanes 1 and 9). Constitutive activation of AKT and ERK signaling pathways are crucial for cell growth and transforming ability of NSCLC cell lines harboring mutant *EGFR* [49, 50]. Consistent with previous reports [51], we found that PI3K/AKT inhibitor NVP-BEZ235 effectively suppressed the growth of both parental PC9 and resistant cell lines in a dose dependent manner, while MEK inhibitor AZD6244 displayed no effect on cell viability (Figure 3C and Supplementary Figure S3C). These results indicate that inhibition of PI3K/AKT signaling pathways resulting from SCRN1 suppression may be directly responsible for increased induction of apoptosis by EGFR-TKIs, resulting in consequent restoration of erlotinib sensitivity in C1 and C2 cells [52, 53]. The growth inhibitory effect of NVP-BEZ235 was increased by combinatorial treatment with erlotinib in parental cells (Figure 3D and Supplementary Figure S4A), but was not observed in C1 and C2 cells (Figure 3E and Supplementary Figure S4B, S4C and S4D) [51, 54]. Given that the levels of SCRN1 are unaffected by erlotinib treatment in these cells, these data provide further evidence that SCRN1 overexpression attenuates the effect of erlotinib on the downstream PI3K/AKT signaling pathway, thereby decreasing the sensitivity to EGFR inhibitory drugs. We found a synergistic growth inhibition effect by coupling NVP-BEZ235 treatment with silencing of *SCRN1* by shRNA in C1 and C2 cells (Figure 3F and Supplementary Figure S4E and S4F), as well as more effective inhibition of PI3K/AKT signaling pathways by NVP-BEZ235 when SCRN1 is downregulated (Supplementary Figure S4G). Clinical significance of these combinatorial response needs further investigated using *in vivo* mouse model in future studies.

While our data from the loss-of-function based approaches provide compelling evidence that show a relationship between the downregulation of *SCRN1* and the restoration of sensitivity to EGFR-TKIs in the resistant clones, we were unable to establish resistance to EGFR-TKIs in any additional *EGFR* mutation-bearing lung cancer cell lines through ectopic overexpression of SCRN1 (Supplementary Figure S5A, S5B and data not shown). The functional uncertainty of SCRN1 mediated resistance in other EGFR mutant backgrounds may reflect differences in genomic alterations, expression profiles and signaling networks that remains unclear at this time.

### Suppression of SCRN1 increases EGFR-TKIs sensitivity for T790M-bearing NCI-H1975 cells by enhancing apoptosis via PI3K/AKT signaling pathway

**Figure 4:**
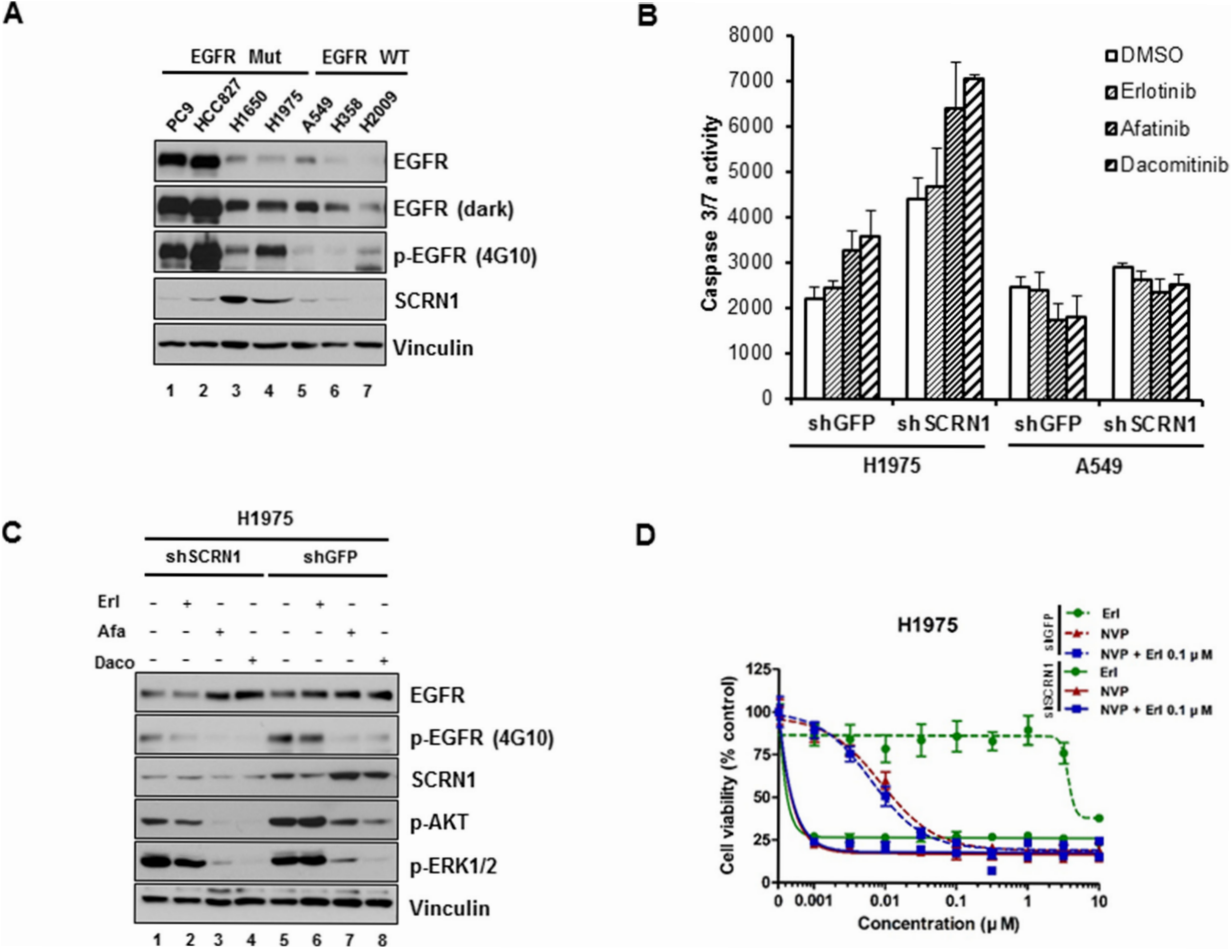
Silencing of *SCRN1* by shRNA significantly increased apoptosis induced by EGFR TKIs in T790M-bearing NCI-H1975 cells (**A**) Levels of SCRN1 protein in various lung adenocarcinoma cell lines were examined by immunoblotting analysis. (**B**) Caspase 3/7 activities induced by EGFR-TKIs in NCI-H1975 cells, but not in A549 cells, were significantly enhanced following *SCRN1* knockdown compared to sh*GFP* control. Values are the means + SD from three independent experiments. (**C**) Levels of phospho-AKT were synergistically diminished by EGFR-TKIs treatment in NCI-H1975 cells expressing shSCRN1. (**D**) Gwoth of H1975 cells are synergistically inhibited by treatment of NVP-BEZ235 and/or erlotinib following shSCRN1 transfection.

We next examined expression of SCRN1 in additional lung adenocarcinoma cell lines by immunoblotting analysis and found that protein levels of SCRN1 were the most elevated in NCI-H1975 and NCI-H1650 cell lines (Figure 4A). NCI-1975 cells are known to be resistant to erlotinib as a consequence of secondary *EGFR* T790M mutation [15, 55], we therefore sought to explore the functional significance of SCRN1 in these cells. Similar to PC9/CYF10 derived C1 and C2 clones, suppression of *SCRN1* via shRNA significantly increased caspase 3/7 activity in NCI-H1975 cells, and treatment with EGFR-TKIs including erlotinib, afatinib or dacomitinib further enhanced apoptosis (Figure 4B). In contrast, A549 cells remained unaffected under the same condition. Consistent with these results, immunoblotting analysis showed that AKT phosphorylation in response treatment with afatinib or dacomitinib was significantly reduced following *SCRN1* suppression by shRNA in NCI-H1975 cells (Figure 4C), demonstrating that suppression of SCRN1 expression enhanced the drug sensitivity of NCI-H1975 cells by modulating PI3K/AKT signaling pathways. The growth of these cells are also significantly abrogated by PI3K/AKT inhibitor NVP-BEZ235, but not by MEK inhibitor AZD6244 (Supplementary Figure S6A) [53]. In addition, similar to PC9/CYF10 erlotinib resistant cells, this growth inhibition by NVP-BEZ235 were significantly increased with suppression of SCRN1 expression, but not by combinatorial treatment with erlotinib (Figure 4D and Supplementary Figure S6B) [56]. In contrast, we did not observe any differences in A549 cells under the same experimental condition (Supplementary Figure S6C). Taken together, these results suggest that upregulation of SCRN1 contributes to cell survival of T790M-mediated erlotinib resistant cells by modulating PI3K/AKT signaling pathways as well.

Furthermore, we analyzed publicly available Cancer Cell Line Encyclopedia (CCLE) data [57] and found that IC_50_ of erlotinib is significantly lower in cell lines with low SCRN1 expression than in cell lines with higher SCRN1 expression (Supplementary Figure S7), supporting the notion that level of SCRN1 expression is correlated with erlotinib sensitivity. However, it needs to be further investigated whether suppression of SCRN1 is directly associated with the growth inhibition of lung cancer cell lines with elevated SCRN1 expression and whether it modulates the sensitivity to EGFR-TKIs in these cell lines.

### SCRN1 overexpression was detected in a subset of EGFR TKIs-resistant NSCLC patients

**Figure 5:**
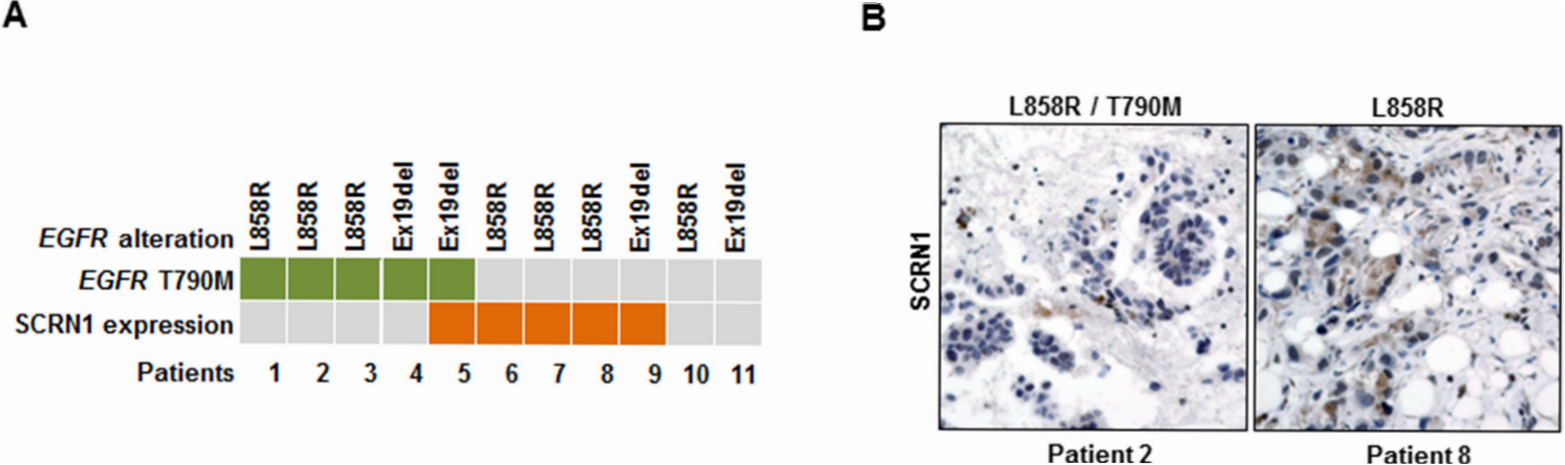
Increased SCRN1 levels were detected in a subset of patient specimens from EGFR-TKIs resistant lung adenocarcinoma patients (**A**) Schematic summary of 11 primary tumor specimens obtained from patients with acquired EGFR-TKI resistant lung adenocarcinoma for the status on T790M mutation in *EGFR* and SCRN1 protein expression determined by immunohistochemistry. (**B**) Immunohistochemical staining for SCRN1. Representative images from specimens (patient 2 and patient 8) that show negative and positive SCRN1 immunohistochemical staining, respectively.

To further investigate the clinical significance of our findings, we measured the levels of SCRN1 by immunohistochemistry (IHC) in 11 tumor specimens from lung adenocarcinoma patients who developed resistance to erlotinib or gefitinib. All analyzed tumor specimens harbored an *EGFR* mutation encoding L858R or the Ex19Del (Supplementary Table S5). Consistent with previous reports [21, 24, 25], we detected *EGFR* T790M gatekeeper mutation in 5 of 11 (45.5%) (Figure 5A), while *MET* amplification measured by fluorescence *in situ* hybridization (FISH) analysis was not detected in any of the specimens tested (Supplementary Table S5). We observed high levels of SCRN1 in 5 out of 11 (45.5%) of the EGFR TKIs-resistant specimens (Figure 5A and 5B). Because we were unable to evaluate the matched pretreatment samples, we cannot exclude the possibility that high SCRN1 expression preexisted in these tumors before EGFR-TKIs treatment. Nevertheless, four of the EGFR-TKIs resistant tumors with detected SCRN1 levels harbored neither an *EGFR* T790M nor amplified *MET*, showing a trend toward mutual exclusivity of SCRN1 expression with *EGFR* T790M or *MET* amplification, however, a larger cohort would be necessary to make a strong statistical conclusion. These results demonstrate that SCRN1 is overexpressed in a subset of activating *EGFR* mutation-bearing lung adenocarcinomas with acquired resistance to EGFR-TKIs. Taken together, we propose that the inhibition of upregulated *SCRN1* in combination with EGFR-TKIs can be used as a novel therapeutic strategy in the treatment of lung adenocarcinoma patients refractory to TKIs.

## MATERIALS AND METHODS

### Expression constructs

pBabe-puro plasmid encoding *EGFR* Ex19Del/CYF10 (Y978F, Y998F, Y1016F, Y1069F, Y1092F, Y1110F, Y1125F, Y1138F, Y1172F, Y1197F) mutant was generated by a series of site-directed mutagenesis reactions with *EGFR* L747_E749del, A750P (Ex19Del) mutation in pBabe-puro as a template. pLX304 *SCRN1* expression plasmid was obtained from The ORFeome Collaboration (Dana-Farber Cancer Institute). All plasmids were sequence verified.

### Cell culture and reagents

NCI-H1975, NCI-H358, NCI-H2009 and A549 cell lines were purchased from the American Type Culture Collection, and NCI-H1650 and HCC827 cells were obtained from Korean Cell Line Bank. PC9 cells were kindly provided by Matthew Meyerson (Harvard Medical School). Transient transfection experiments were performed using XtremeGene 9 (Roche) according to manufacturer's instruction. Cells were serum-starved for 16 hours before drug treatment and harvested for making lysates. Erlotinib (LC laboratories), afatinib (LC laboratories), dacomitinib (Selleckchem), NVP-BEZ235 (Selleckchem, PI3K/AKT inhibitor) and AZD 6244 (Selleckchem, MEK inhibitor) were dissolved in DMSO at 10 mM.

### Anchorage-independent growth assay

Soft agar assays were conducted in triplicated as previously described (Cho et al., 2013) with minor changes (2 × 10^4^ cells were used per well). After 2–3 weeks, digital images were taken and the number of colonies was quantified using GelCount software (Oxford) according to the manufacturer's protocol. The data were presented as a relative ratio in a graph following normalization to number of colonies formed by control cells. Each assay was repeated a minimum of two times with comparable results.

### Cell growth inhibition assay

PC9 (8 × 10^3^ cells), NCI-H1975 (8 × 10^3^ cells) or A549 (6 × 10^3^ cells) were plated in 180 μl media in 96-well flat-bottom plates (Corning). After 24 hours, cells were treated with drugs at the indicated concentrations and incubated as previously described [58]. For the RNAi study, cells were seeded into 96 well plates for overnight and then transfected with shRNA expressing lentiviruses with polybrene for 72 hours. Transfected cells were treated with indicated drugs for additional 72 hours. Viable cell numbers were measured using Cell Counting Kit-8 solution (Dojindo). Absorbance was measured at 450 nm after 3 hours. Data are expressed as percentage of growth relative to that of untreated control cells.

### Immunoblotting and antibodies

Cells were lysed in RIPA buffer supplemented with protease inhibitors (Roche) and phosphatase inhibitor cocktail II and IV (Calbiochem) and subjected to immunoblotting. Antibodies against pAKT, pERK1/2 and β-actin were purchased from Cell Signaling Technology. Anti-EGFR, anti-SCRN1 and 4G10 antibodies were purchased from Bethyl, Abcam and Millipore, respectively. Antibodies against Vinculin were purchased from Sigma.

### RNAi studies

pLKO.shRNA plasmids targeting *SCRN1* and *GFP* were purchased from Sigma and viruses were produced using protocols from the RNAi Consortium (http://www.broadinstitute.org/rnai/trc). Cells were plated one day prior to infection and subsequently incubated with diluted virus containing media with 8 μg/mL polybrene for 4 hours. Transfected cells were pooled and treated with puromycin for 1 week. For siRNA studies, cells plated in 10cm plate or 6-well plates were transfected with either *SCRN1* targeting siRNA (Bioneer) using Lipofectamine (Invitrogen) according to the manufacturer's instructions. Scrambled siRNA (Dharmacon) used as negative control. After 48 hours, transfected cells were treated with drugs as indicated followed by immunoblotting.

### Caspase 3/7 activity assay

Caspase 3/7 activity in cell extracts and culture supernatant was measured using the Caspase-Glo 3/7 Assay Kit (Promega) according to manufacturer's instruction. Briefly, parental or transfected cells were plated in 96-well plate and treated with either vehicle, erlotinib, afatinib or dacomitinib for 24 hours. After addition of 100 μl Caspase 3/7 reagent and a gentle mixing, the cells were incubated for 1 hour at room temperature, and the luminescence of each sample was measured by luminometer.

### Xenografted mouse study

All animal experiments were carried out in accordance with IACUC of Laboratory Animal Research Center (LARC; AAALAC International-approved facility) in Samsung Medical Center. PC9 and resistant cell lines (10^7^/50 μl) were injected into 4 week-old male BALB/c-nude mice (*n* = 4 or 5 mice per group). Mice were purchased from Charles River Japan. Mice were randomly assigned to erlotinib treatment or no treatment groups for each cell line. After the tumor size reached approximately 150–300 mm^3^, mice were treated with erlotinib (25 mg/kg/day) or control three times per week by oral gavage. Tumor volume was measured using caliper three times per week and calculated using the formula *Volume = Length × Width*^2^/*2*. Mice were sacrificed when morbid.

### Immunohistochemistry

Immunohistochemistry for SCRN1 was performed on unstained slides using antibody to SCRN1 (rabbit polyclonal, dilution 1:50, Abcam). Slides were cut to a thickness of 4 μm, deparaffinized in xylene and hydrated in a graded series of alcohols. Heat-induced antigen retrieval was performed by using a microwave oven and ER 1 buffer (pH 6.0). Sections were incubated in Bond-max autoimmunostainer (Leica Biosystem) for 20 minutes at 97°C. The IHC reactions were visualized using Bond-max autoimmunostainer (Leica Biosystem) using Bond™ Polymer refine detection, DS9800 (Vision Biosystems). For SCRN1 immunohistochemistry, only cytoplasmic staining was evaluated and the case with more than 10% of positive staining cancer cells was counted as positive.

### Fluorescence *in situ* hybridization (FISH) analysis

Deparaffinized 4 μm sections were submitted to dual-color FISH analysis according to the manufacturer's instructions using a Histology FISH Accessory Kit (Vysis, Illinois, USA) with a *MET*/CEN7q Dual Color FISH Probe purchased from Abbot laboratory (Vysis, Illinois, USA). Results are interpreted as the average ratio of *MET*/chromosome 7 signals in 50 non-overlapping nuclei.

### Quantitative real-time PCR analysis

Total cellular RNA was prepared from the cells by using and RNeasy Mini Kit (Qiagen). And total RNA was used to synthesize the first strand cDNA using RNA to cDNA EcoDry premix Oligo-dT (Clontech). Quantitative PCRs were performed with the use of SYBR green PCR Master Mix (Toyobo) [59] and we used an ABI 7300 real time PCR system (Applied Biosystems). GAPDH was used as the internal standard for normalization. The primer sequences used are as follows; SCRN1 primer set (forward: 5′-GGAGAGGGCGAGTTCAATTT-3′; reverse: 5′-GCACTGTGATGCTTTCTTCTTG-3′), GAPDH primer set (forward: 5′-GGTGTGAACCATGAGAAGTATGA-3′; reverse: 5′-GAGTCCTTCCACGATACCAAAG-3′).

### RNAseq-based gene expression profiling

Total RNA purified from two independent cultures of DMSO passaged control and erlotinib resistant cultures of PC9 cells was used to prepare cDNA for sequencing with the Illumina HiSeq2500 using the TruSeq RNA sample preparation kit (Illumina, USA). Paired-end 50 base pair RNA-sequencing reads were mapped to the human RefSeq transcripts using DNAnexus (http://dnanexus.com) analysis module and gene expression values were obtained by the RPKM normalization method. We analyzed the data with use of the samr (Significance Analysis of Microarray in R) package in Bioconductor. After the genes whose RPKM is < 10 in all samples were filtered out, genes with fold change > 50% with absolute RPKM change > 10 with a statistical SAM score exceeding expected value at a delta slope of 2.5 were considered statistically significant as differentially expressed. We excluded genes whose expression in RPKM increased by > 2-fold upon treatment with erlotinib from the above identified upregulated genes. Those genes whose expression is significantly upregulated or downregulated in resistant PC9 cells were also used for Gene Set Enrichment Analysis (GSEA) against Molecular Signature Database (MSigDB) v3.0.

### siRNA library screening

Four siRNA sequences for each targeted gene were picked from the Whole Human Genome siRNA Library (Qiagen) to create custom 384-well assay plates. Total of 320 siRNAs against 80 up-regulated genes were picked. In all assay plates, we included negative control siRNAs (Non-Silencing, All-Star Non-Silencing, and GFP, Qiagen), and two positive control siRNAs for transfection (UBBs1 and All-Star Cell Death Control, Qiagen). The siRNAs were printed individually into white solid 384-well plates (1l of 0.667 μM siRNA per well for a total of 9 ng siRNA) using a Biomek FX (Beckman Coulter). Lipofectamine was transferred into each well. Cells were added into each well using a BIO-TEK mFill Microplate Dispenser. Transfected cells were incubated for 24 hours prior to the addition of 7.5 nM Erlotnib for sensitive parental cells and 2 mM erlotinib for resistant cells as well as vehicle control (DMSO). After further incubation for additional 72 hours, cell viability was measured using an Analyst GT Multimode reader (Molecular Devices). Each condition was run in duplicate for all cell lines. A biological replicate of the screen was also performed for all cell lines, thus 4 replicates per condition for each cell line.

### RNAi screening data analysis

The siRNA screen included parental and resistant cell lines, which were either untreated and treated with Erlotinib and each cell line had 2 technical and 2 biological runs. The output of the raw luminescence data from the plate readers were aligned and annotated with their respective gene names and control types. The output from ratio normalization was then used as an input to RNAi gene enrichment ranking algorithm (RIGER) [38]. RIGER, a java extension of GENE-E software package (http://www.broadinstitute.org/cancer/software/GENE-E) was used to calculate for enrichment of multiple siRNAs targeting the same gene. Signal to noise metric for ranking siRNAs and Kolmogorov-Smirnov method was used to convert individual siRNA to genes. RIGER methodology is nonparametric in its approach and uses Gene Set Enrichment Analysis (GSEA) and Kolmogorov–Smirnov (KS) to calculate gene scores from multiple siRNAs targeting a gene. A list of ranked genes with Normalized Enrichment Scores (NES) [60] is generated by RIGER.

### SCRN1 expression and erlotinib sensitivity analysis

We downloaded CCLE anti-cancer drug sensitivity and gene expression data for human tumor cell lines (http://www.broadinstitute.org/ccle). The downloaded gene expression profile was pre-processed by z-score transformation across samples. To measure the association between the gene expression level of *SCRN1* and response to erlotinib, IC_50_ values for erlotinib in cell lines were compared between two groups, high SCRN1-expressing and low expressing cell lines (using a cutoff value = 0.5). The significance of difference between IC50 values were compared using the Student's two sample *t*-test.

## SUPPLEMENTARY MATERIALS FIGURES AND TABLES






