# Supplementary material for: Integrated genomic approaches identify upregulation of SCRN1 as a novel mechanism associated with acquired resistance to erlotinib in PC9 cells harboring oncogenic EGFR mutation
Source: Oncotarget. 2016 Feb 11;7(12):13797–809. doi: 10.18632/oncotarget.7318 (PMC4924679; doi:10.18632/oncotarget.7318)
Supplement: Supplementary file 1 [file oncotarget-07-13797-s001.pdf]

Integrated genomic approaches identify upregulation of SCRN1 as a novel mechanism associated with acquired resistance to erlotinib in PC9 cells harboring oncogenic EGFR mutation

Supplementary Materials

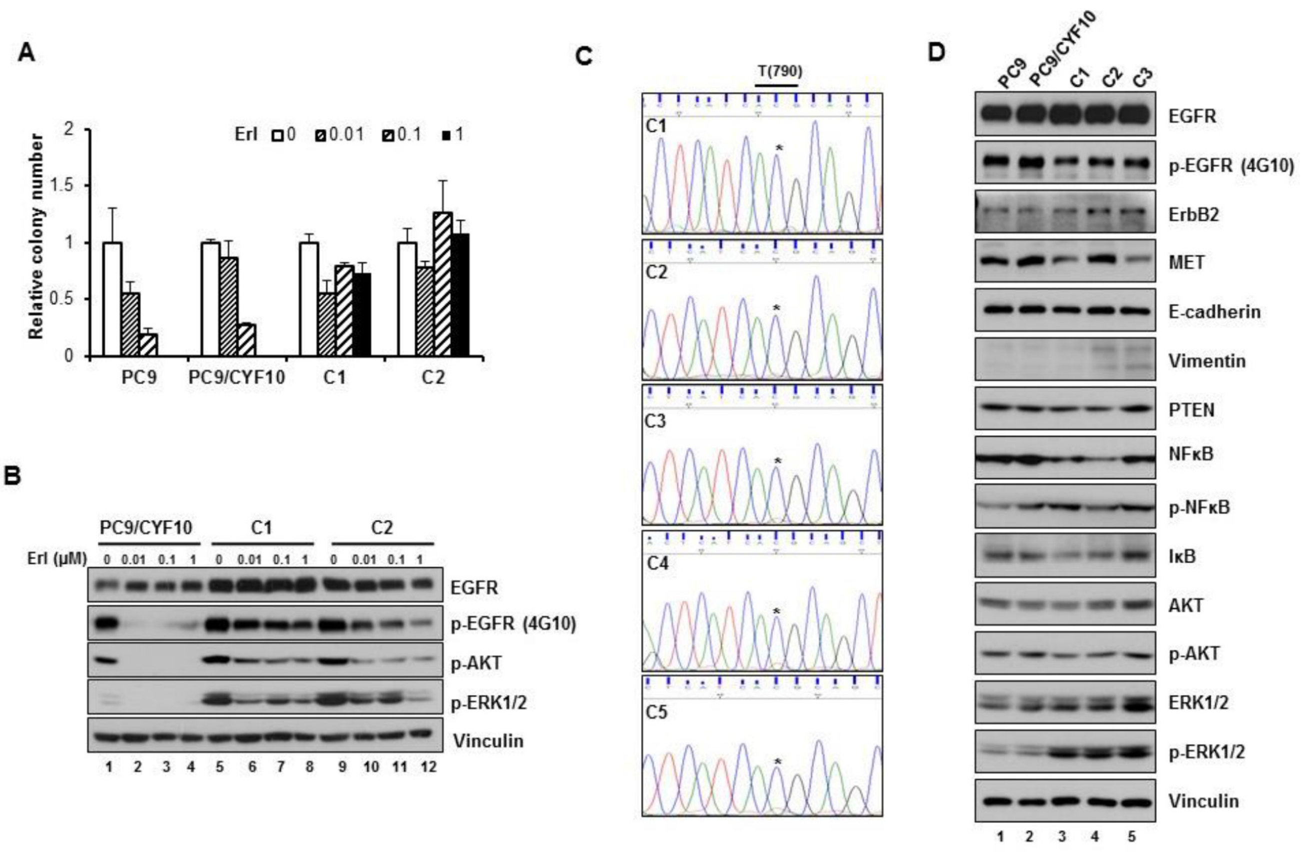

Supplementary Figure S1: Characterization of EGFR-TKIs resistant clones. (**A**) Cell growth of C1 and C2 resistant cells was not inhibited by even high dose of erlotinib treatment. Parental cells, C1 and C2 clones were assayed for anchorage-independent growth in soft agar in the presence of erlotinib as indicated. The bar graph depicts the relative number of colonies in parental or resistant clones treated with erlotinib normalized to the number of colonies by cells without erlotinib treatment ($n = 3$, mean + SD). (**B**) In contrast to parental cells, the basal levels of phospho-EGFR, phospho-AKT and phospho-ERK1/2 in C1 and C2 cells were not completely blocked by erlotinib treatment as shown by immunoblotting analysis. Vinculin was used as a loading control. (**C**) *EGFR* T790M mutation was not found in any of the EGFR-TKIs resistant clones (C1 to C5 clones). Sequencing chromatograms of EGFR exon 20 region encompassing Thr790 residues were shown. (**D**) No differences of previously known protein expression associated with EGFR-TKIs were observed in erlotinib resistant PC9/CYF10 clones by immunoblotting analysis. Cell lysates prepared from PC9, PC9/CYF10, C1, C2 or C3 cells were subjected to immunoblotting analysis with the indicated antibodies. Vinculin served as a loading control.

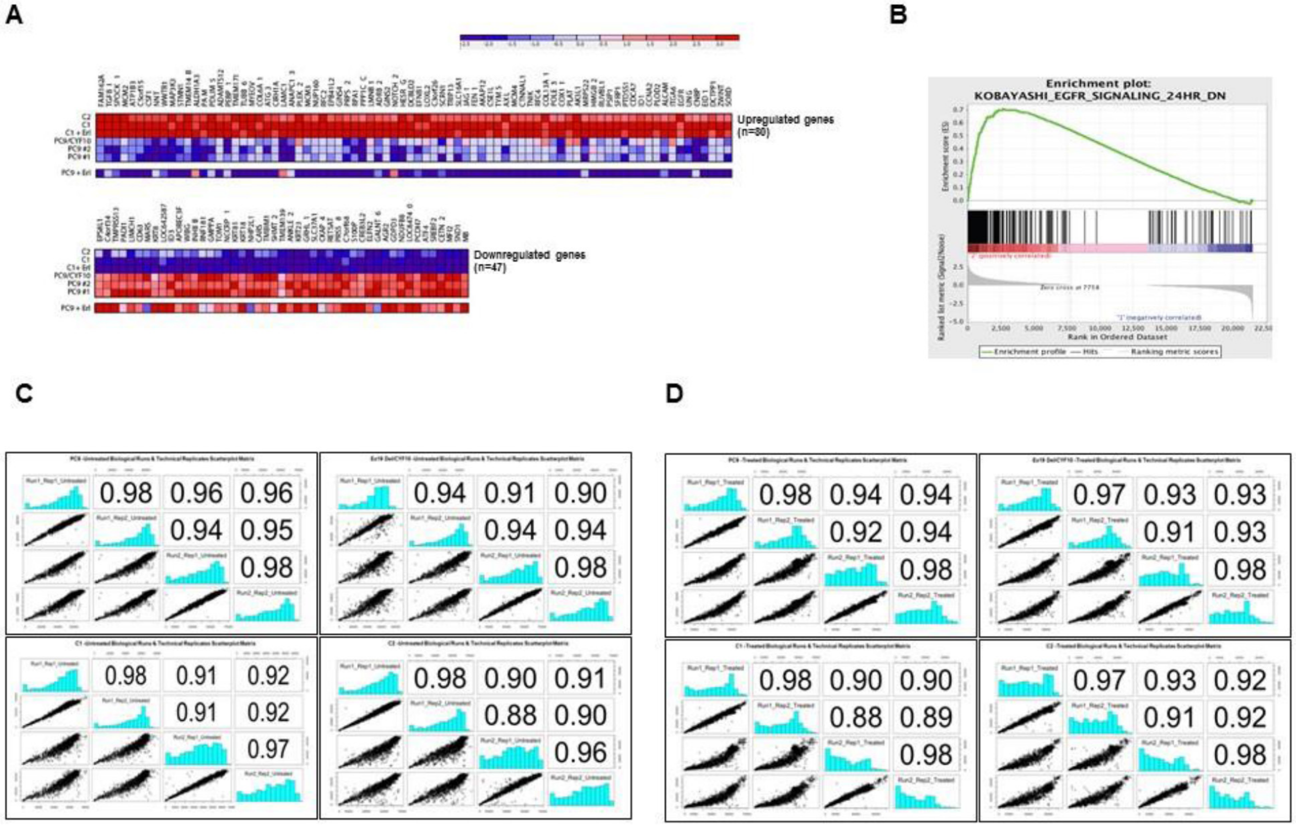

Supplementary Figure S2: Candidate genes identified by RNAseq-based expression profiling for siRNA synthetic lethality screening. (**A**) Heatmap of RNA based gene expression profiling indicates significant differential expression of specific gene in parental and EGFR-TKI resistant cell lines. Red color indicated that specific up-regulation genes and blue color indicates specific down-regulation genes in resistant cell lines. (**B**) Gene set enrichment analysis (GSEA) for genome wide expression profiles of common biological function revealed that downregulated genes are significantly correlated with the expression profiles generated in NCI-H1975 cells. (**C**) and (**D**). Scatterplots of biological runs and technical replicates for siRNA synthetic lethality screens with parental and resistant clones with elrotinib treated (D) or untreated (C). Data indicate that the replicates were highly reproducible (majority R^2 > 0.9) and well maintained throughout the experiments.

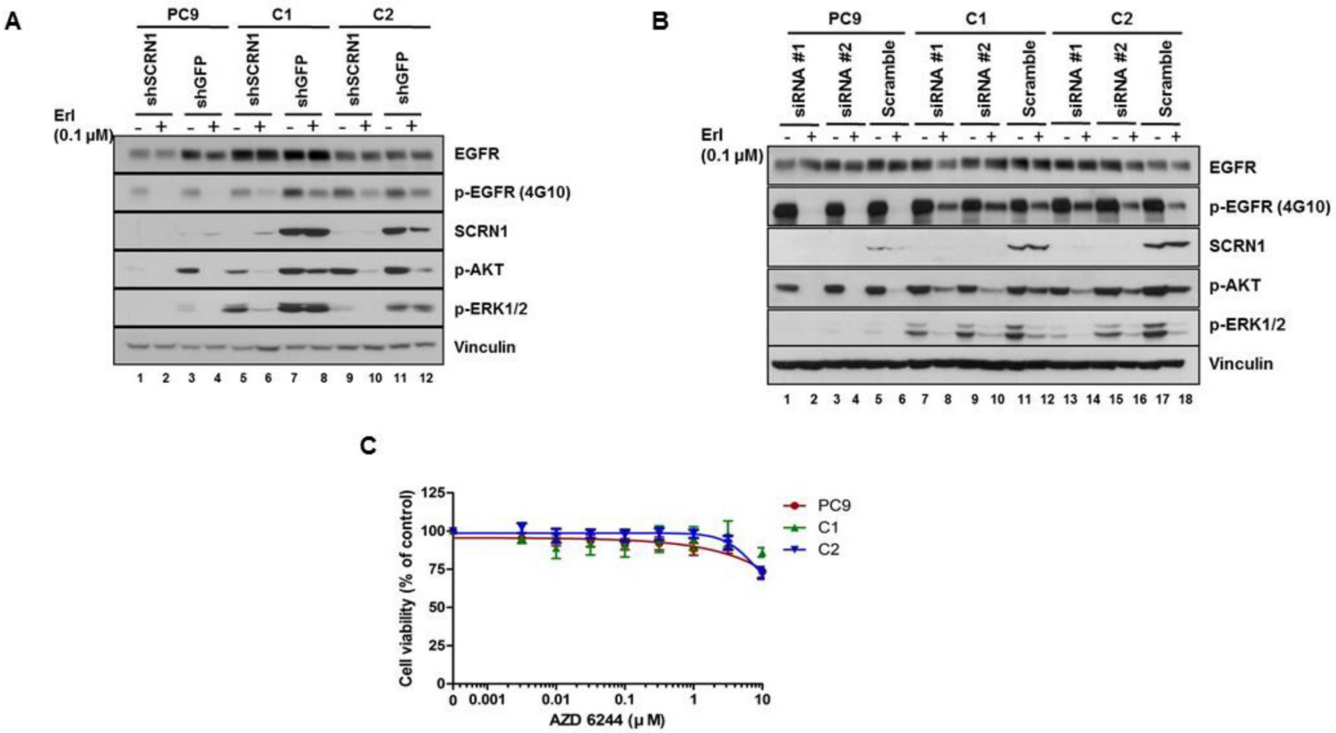

Supplementary Figure S3: Levels of constitutively phosphorylated AKT and ERK1/2 were more robustly reduced by erlotinib in C1 and C2 cells infected with sh*SCRN1* than in those with sh*GFP*. (**A**) Cell lysates prepared from PC9, C1 or C2 cells expressing either sh*SCRN1* or sh*GFP* treated or untreated with erlotinib for 3 hours, were subjected to immunoblotting analysis with the indicated antibodies. Vinculin was used as a loading control. (**B**) PC9, C1 or C2 cells transfected with *SCRN1* targeting siRNA #1 or #2 were incubated for 3 hour with or without erlotinib, and the resulting lysates were subjected to immunoblotting analysis with the indicated antibodies. Vinculin was used as a loading control. (**C**) Cell viability of PC9, C1 or C2 cells were unaffected by treatment of MEK inhibitor AZD6244. PC9, C1 and C2 cells exposed to increasing concentrations of the MEK inhibitor AZD6244. The results are presented as a mean ± SD of sextuplicate wells and are representative of three independent experiments.

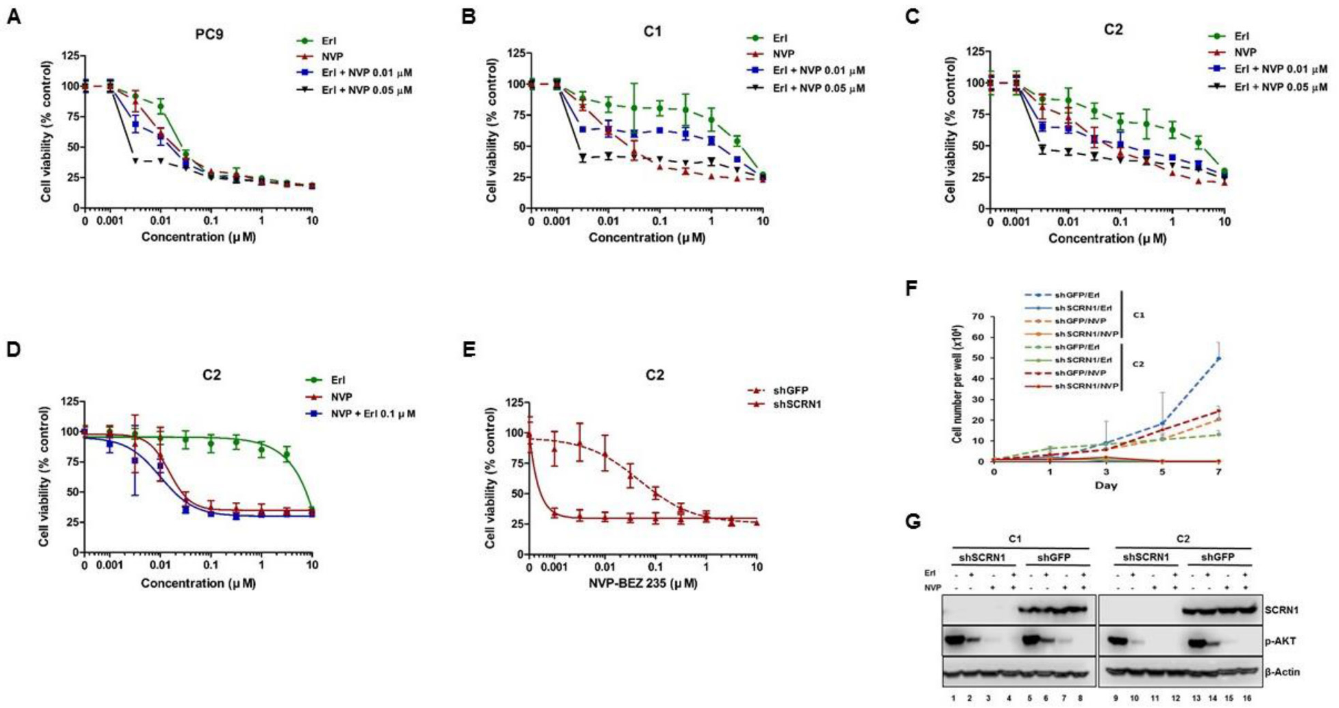

Supplementary Figure S4: Suppression of SCRN1, but not elrotinib treatment, synergistically increased the growth suppression effect of NVP-BEZ235 in erlotinib-resistant cell lines. (**A**, **B** and **C**) NVP-BEZ235 synergistically increased the sensitivity of erlotinib for PC9 cell (A), but not for both C1 (B) and C2 cells (C). The results are presented as a mean ±SD of sextuplicate wells and are representative of three independent experiments. (**D** and **E**) Growth inhibition of C2 cell clone by NVP-BEZ235 is unaffected in the presence of erlotinib treatment (D), but dramatically increased in combination with shRNA-mediated silencing of *SCRN1* (E). The results are presented as a mean ±SD of sextuplicate wells and are representative of three independent experiments. (**F**) Cell proliferation of C1 and C2 clones are effectively inhibited by either downregulation of *SCRN1* alone or in combination with NVP-BEZ235 or erlotinib. (**G**) Immunoblotting analysis showing that levels of p-AKT further diminished by co-treatment of NVP-BEZ235 and erlotinib in the presence of shSCRN1 in C1 and C2 cells. Beta-actin used as a loading control.

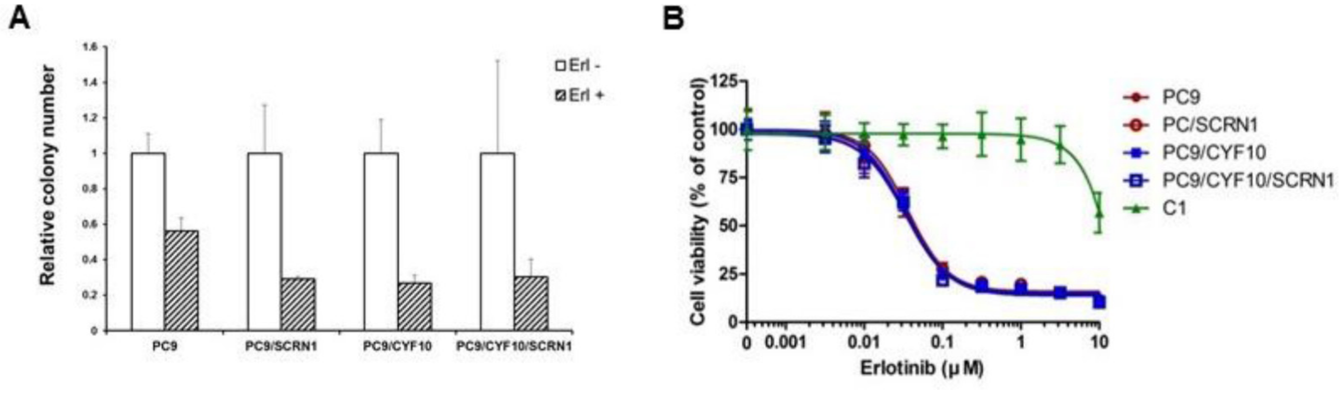

Supplementary Figure S5: Ectopic expression of SCRN1 in PC9 and PC9/CYF10 cells did not render the resulting cells to be resistant to erlotinib. (**A**) Anchorage independent growth of PC9 and PC9/CYF10 cells expressing SCRN1 was measured by colony formation assay in soft agar in the presence or absence of erlotinib as indicated. The bar graph depicts the relative number of colonies in parental or SCRN1 overexpressing cell lines treated with erlotinib normalized to the number of colonies by cells without erlotinib treatment (*n* = 3, mean + SD). (**B**) Cell viability of PC9 and PC9/CYF10 cells expressing SCRN1 are equally sensitive to erlotinib as the parental cell lines. PC9, PC9/CYF10, PC9/SCRN1, PC9/CYF10/SCRN1 or C1 cells exposed to increasing concentrations of erlotinib as indicated for 72 hours were assayed for cell viability using Cell Counting Kit-8 reagents. The results are indicated as mean ± SD of three independent experiments.

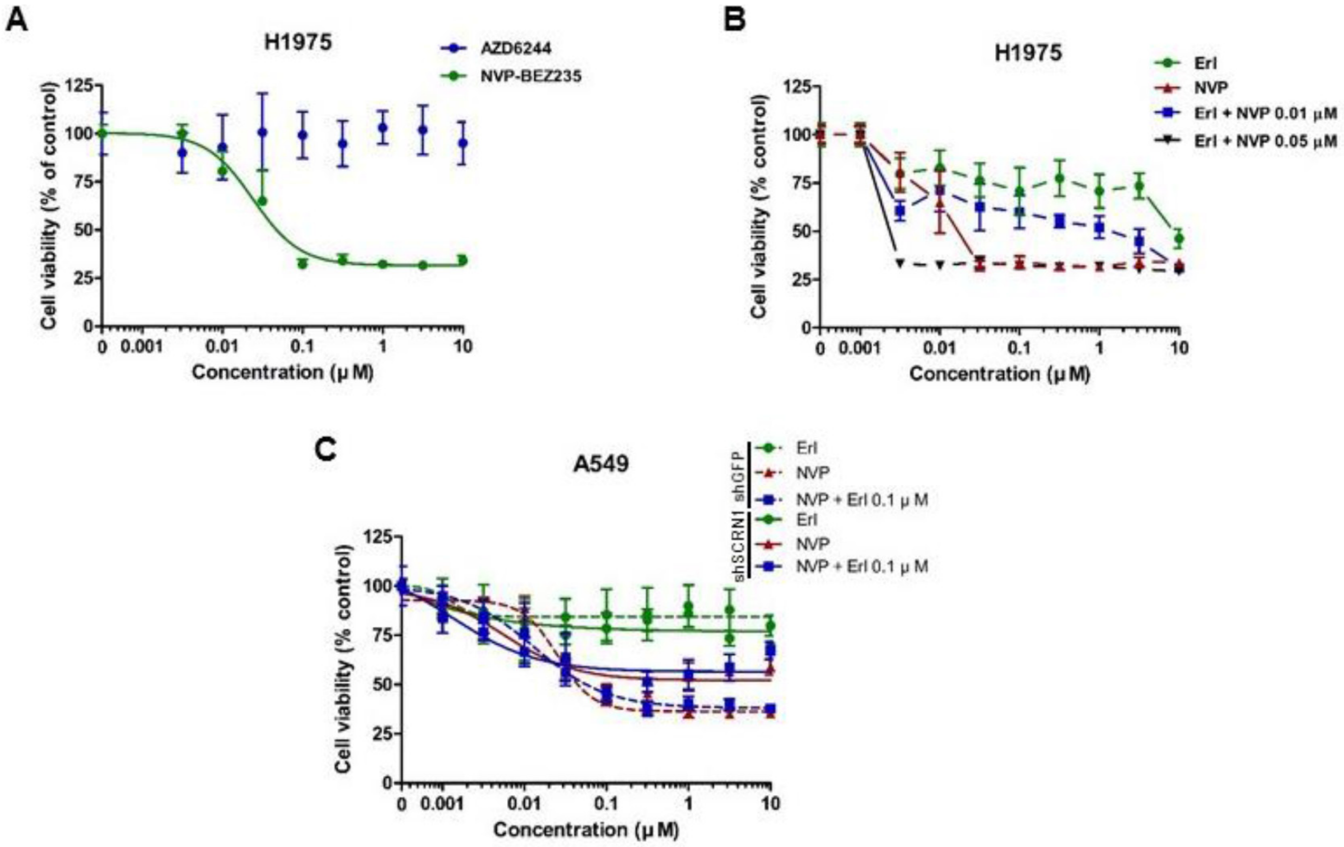

Supplementary Figure S6: Growth inhibition of NCI-H1975 cells by NVP-BEZ235 is not synergistic with erlotinib. (**A**) NCI-H1975 cells treated with either AKT inhibitor (NVP-BEZ235) or MEK inhibitor (AZD6244) at the indicated concentrations for 72 hours and assayed for cell viability using Cell Counting Kit-8 (CCK-8). (**B**) The sensitivity of erlotinib for NCI-H1975 cells is unaffected in the presence of NVP-BEZ235. (**C**) Growth suppression of A549 cells by NVP-BEZ235 was not enhanced by cotreatment with erlotinib or silencing of *SCRN1*.

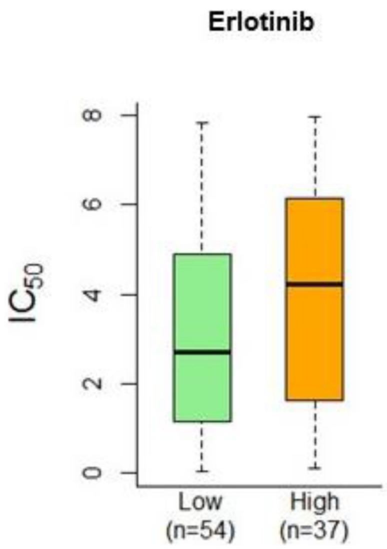

Supplementary Figure S7: The expression levels of *SCRN1* are anti-correlated with the IC_{50} value to erlotinib in cancer cell lines. The relationship between *SCRN1* expression level and response to erlotinib were examined by comparing log10 half maximum inhibitory concentration (IC_{50}) values for erlotinib in cell lines between high *SCRN1*-expressing (n = 37) and low expressing (n = 54) cell lines (data obtained from CCLE). We downloaded CCLE anti-cancer drug sensitivity and gene expression data for human tumor cell lines (http://www.broadinstitute.org/ccle). The downloaded gene expression profile was pre-processed by z-score transformation across samples. To measure the association between the gene expression level of *SCRN1* and response to erlotinib, IC_{50} values for erlotinib in cell lines were compared between two groups, high SCRN1-expressing and low expressing cell lines (using a cutoff value = 0.5). The significant difference was observed between two groups (p = 0.05), which were compared using the Student's t-test.

Supplementary Table S1 : Analyzed SAM score for all genes

Supplementary Table S2 : Up and down-regulated genes selected by SAM

Supplementary Table S3: List of genes that upon knockdown showed statistical significance (*p*-value < 0.05) between the group comparisons

Gene rank	Treated Parental vs Treated Resistant			Untreated Resistant vs Treated Resistant			Untreated Parental vs Untreated Resistant		
	**Gene**	**NES**	***p*-value**	**Gene**	**NES**	***p*-value**	**Gene**	**NES**	***p*-value**
1	MRPS22	2.04	0	NUP160	1.8787	0.0003	TNIK	1.7044	0.0072
2	MYEOV	1.8957	0	EPB41L2	1.8386	0.0008	MRPS22	1.6704	0.009
3	EFNB1	1.8209	0.0004	ATP1B3	1.8147	0.0014	***SCRN1***	1.6743	0.009
4	MCM4	1.7789	0.0011	TMEM14B	1.7819	0.0019	PLOD2	1.6517	0.01
5	LAMC1	1.7443	0.0016	MRPS22	1.7665	0.0023	CCNA2	1.6491	0.0102
6	ANAPC13	1.6503	0.0042	MYEOV	1.7115	0.0036	ADRB2	1.6399	0.0105
7	SLC16A1	1.6424	0.0043	PLEK2	1.5918	0.0079	NUP160	1.5839	0.0136
8	TMEM14B	1.6129	0.0055	LAMC1	1.5459	0.0105	MYEOV	1.5697	0.0143
9	DCBLD2	1.6029	0.006	TNIK	1.529	0.0114	PTDSS1	1.503	0.0189
10	***SCRN1***	1.5478	0.0079	ZNF9	1.5171	0.0117	ANAPC13	1.4675	0.0223
11	ADAMTS12	1.4637	0.0126	CRI1	1.5042	0.0125	MCM4	1.4238	0.0269
12	PPP1CC	1.4168	0.016	PSIP1	1.4908	0.0131	EPB41L2	1.4215	0.027
13	PSIP1	1.3818	0.0193	XTP3TPA	1.4101	0.0199	DCBLD2	1.4045	0.0287
14	APG3L	1.3673	0.0205	AK3L1	1.4041	0.0205	PAM	1.3865	0.0307
15	PBP	1.3571	0.0222	LMNB1	1.4006	0.0212	PRPS2	1.3818	0.0311
16	EGFR	1.3463	0.0228	***SCRN1***	1.3846	0.0228	POLE3	1.3746	0.032
17	TNIK	1.3407	0.0234	NNT	1.3686	0.0247	SLD5	1.3692	0.0324
18	PRPS2	1.3445	0.0234	MCM4	1.3652	0.0248	SLC16A1	1.3514	0.0345
19	PDLIM5	1.3322	0.0247	JAG1	1.3625	0.0252	AXL	1.3264	0.0384
20	NNT	1.3365	0.0247	COL13A1	1.3293	0.0281	TMEM14B	1.277	0.0471
21	CCNA2	1.3307	0.0248	TRIP13	1.287	0.033			
22	PLAT	1.3256	0.0252	CCNA2	1.2505	0.0387			
23	ATP1B3	1.2973	0.0281	MCM2	1.2546	0.0387			
24	EPB41L2	1.2898	0.0291	PBP	1.256	0.0387			
25	CSE1L	1.2885	0.0299	CDCA7	1.2433	0.0397			
26	NUP160	1.2406	0.0351	PLAT	1.2427	0.0412			
27	RPA1	1.2413	0.0351	SLC16A1	1.2311	0.0422			
28	ZNF9	1.2358	0.0366	MGC4308	1.2074	0.0462			
29	AXL	1.22	0.0387	NOTCH2	1.1994	0.049			
30	E21G5	1.1718	0.0473						

Supplementary Table S4: Top 10 overlapping genes association with EGFR in lung adenocarcinoma

Gene	TCGA[†]		TCGA pub[††]		Broad[†††]	
	Odds ratio	*p*-value	Odds ratio	*p*-value	Odds ratio	*p*-value
SCRN1	11.33333	0.001327	11.625	0.000128	15.64286	0.000605
CCNA2	4.521739	0.338663	< 0.000001	0.681792	< 0.000001	0.813187
EPB41L2	3.607143	0.119197	1.947368	0.350836	2.25	0.311787
GATA4	2.133333	0.201672	2.218487	0.109603	3.017241	0.070369
NUP160	1.097826	0.649169	1.598291	0.536883	2.212121	0.464315
MCM4	0.717391	0.614164	0.380342	0.303713	2.767742	0.170683
TNIK	0.527174	0.473185	2.234568	0.171546	< 0.000001	0.119188
MRPS22	2.23913	0.46364	< 0.000001	0.463117	< 0.000001	0.813187
SLC16A1	< 0.000001	0.53636	0.948718	0.719949	< 0.000001	0.351048
MYEOV	< 0.000001	0.182925	0.947368	0.652333	< 0.000001	0.433935

[†]TCGA provisional (www.cbioportal.org), [††]Cancer Genome Atlas Research, 2014, [†††]Imielinski et al, 2012. Strong tendency towards mutual exclusivity (0 < Odds ratio < 0.1); Some tendency towards mutual exclusivity (0.1 < Odds ratio < 0.5); No association (0.5 < Odds ratio < 2); Tendency toward co-occurrence (2 < Odds ratio < 10); Strong tendency towards co-occurrence (Odds ratio > 10).

Supplementary Table S5: Upregulated SCNR1 in human EGFR mutant specimens from patients with acquired EGFR-TKI resistance

Patient ID	Age (years)	Sex	EGFR alteration	SCRN1 expression	EGFR T790M	Met amplification[†]	EGFR TKI	PFS (months)	Smoking
1	61	F	L858R	−	+	−	gefitinib	22.4	0
2	77	F	L858R	−	+	−	gefitinib	10.7	1
3	62	F	L858R	−	+	−	gefitinib	10.433	0
4	57	F	Exon 19 del	−	+	−	gefitinib	14.233	0
5	66	M	Exon 19 del	+	+	−	gefitinib	26.167	0
6	54	M	L858R	+	−	−	gefitinib	2.533	2
7	60	M	L858R	+	−	−	gefitinib	7.067	1
8	56	F	L858R	+	−	−	gefitinib	5.6	0
9	68	F	Exon 19 del	+	−	−	gefitinib	28.433	0
10	79	F	L858R	−	−	N/A	gefitinib	N/A	0
11	62	F	Exon 19 del	−	−	−	gefitinib	N/A	0

[*]Clinical characteristics and expression of the indicated biomarkers in the 11 EGFR mutant NSCLC specimens obtained from patients upon acquired resistance to treatment to gefitinib. M, male; F, female; PFS, progression free survival, interval between the beginning of EGFR-TKI and the progression time; 0, never; 1, previous; 2, current smoking; N/A, not enough tissue available for analysis.
